# Bis{μ-2,5-bis­[4-(2-pyridylmethyl­amino)phen­yl]-1,3,4-oxadiazole}bis­[dichlorido­mercury(II)]

**DOI:** 10.1107/S1600536808006788

**Published:** 2008-03-14

**Authors:** Li-Li Liu, Gui-Ge Hou, Jian-Ping Ma, Ru-Qi Huang, Yu-Bin Dong

**Affiliations:** a College of Chemistry, Chemical, Engineering and Materials Science, Shandong Normal University, Jinan 250014, People’s Republic of China

## Abstract

In the title centrosymmetric compound, [Hg_2_Cl_4_(C_26_H_22_N_6_O)_2_], each Hg^II^ center adopts a distorted HgN_3_Cl_2_ trigonal bipyramidal coordination geometry, formed by two pyridine N atoms, one imine N atom and two chloride anions. Within the organic ligand, the oxadiazole ring is nearly coplanar with the two benzene rings [dihedral angles = 5.9 (4) and 6.5 (4)°] and nearly perpendicular to the two pyridine rings with the same dihedral angle of 77.4 (4)°. The two organic ligands bridge two Hg^II^ ions to form the macrocyclic complex. Inter­molecular N—H⋯Cl and N—H⋯N hydrogen bonding helps to stabilize the crystal structure.

## Related literature

For general background, see: Dong *et al.* (2003 ▶). For related structures, see: Gallagher *et al.* (1999 ▶); Grupce *et al.* (1999 ▶). For synthesis, see: Ren *et al.* (1995 ▶).
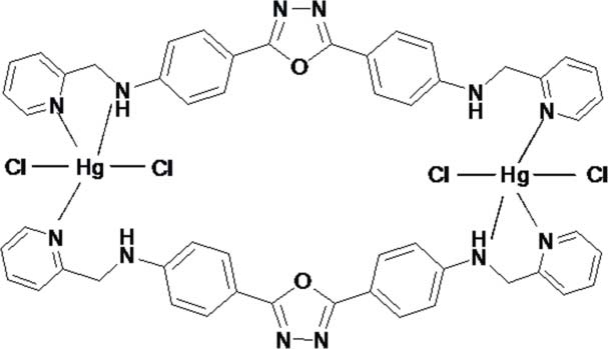


## Experimental

### 

#### Crystal data

[Hg_2_Cl_4_(C_26_H_22_N_6_O)_2_]
*M* *_r_* = 1411.98
Triclinic, 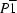

*a* = 8.5426 (19) Å
*b* = 9.945 (2) Å
*c* = 16.533 (4) Å
α = 83.773 (3)°
β = 80.001 (3)°
γ = 67.671 (2)°
*V* = 1278.2 (5) Å^3^
*Z* = 1
Mo *K*α radiation
μ = 6.26 mm^−1^
*T* = 298 (2) K
0.40 × 0.40 × 0.30 mm

#### Data collection

Bruker SMART APEX CCD diffractometer
Absorption correction: multi-scan (*SADABS*; Sheldrick, 2002 ▶) *T*_min_ = 0.113, *T*_max_ = 0.153
6681 measured reflections
4652 independent reflections
3878 reflections with *I* > 2σ(*I*)
*R*_int_ = 0.023

#### Refinement

*R*[*F* ^2^ > 2σ(*F* ^2^)] = 0.044
*wR*(*F* ^2^) = 0.118
*S* = 1.04
4652 reflections
325 parameters
H-atom parameters constrained
Δρ_max_ = 2.20 e Å^−3^
Δρ_min_ = −0.84 e Å^−3^

### 

Data collection: *SMART* (Bruker, 2000 ▶); cell refinement: *SAINT* (Bruker, 2000 ▶); data reduction: *SAINT*; program(s) used to solve structure: *SHELXTL* (Sheldrick, 2008 ▶); program(s) used to refine structure: *SHELXTL*; molecular graphics: *SHELXTL*; software used to prepare material for publication: *SHELXTL*.

## Supplementary Material

Crystal structure: contains datablocks global, I. DOI: 10.1107/S1600536808006788/xu2406sup1.cif

Structure factors: contains datablocks I. DOI: 10.1107/S1600536808006788/xu2406Isup2.hkl

Additional supplementary materials:  crystallographic information; 3D view; checkCIF report

## Figures and Tables

**Table:** 

Hg1—Cl1	2.373 (2)
Hg1—Cl2	2.451 (2)
Hg1—N3	2.587 (6)
Hg1—N4	2.275 (6)
Hg1—N6^i^	2.745 (7)

**Table:** 

N4—Hg1—Cl1	145.19 (16)
N4—Hg1—Cl2	99.31 (16)
Cl1—Hg1—Cl2	114.87 (10)
N4—Hg1—N3	70.81 (18)
Cl1—Hg1—N3	98.44 (13)
Cl2—Hg1—N3	95.30 (13)
N4—Hg1—N6^i^	86.73 (19)
Cl1—Hg1—N6^i^	84.60 (15)
Cl2—Hg1—N6^i^	115.31 (15)
N3—Hg1—N6^i^	144.82 (18)

Symmetry code: (i) 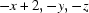.

**Table 2:** Hydrogen-bond geometry (Å, °)

*D*—H⋯*A*	*D*—H	H⋯*A*	*D*⋯*A*	*D*—H⋯*A*
N3—H3⋯N2^ii^	0.91	2.36	3.191 (8)	152
N5—H5⋯Cl1^iii^	0.86	2.68	3.517 (7)	166

Symmetry codes: (ii) 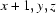; (iii) 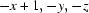.

**Fig. 1.**
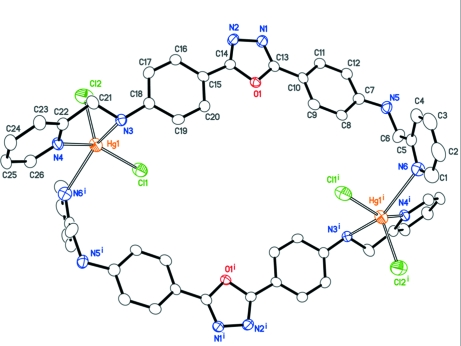
The structure of (I), showing 30% displacement ellipsoids, hydrogen atoms have been omitted [symmetry code: (i) -x + 2,-y,-z].

## supplementary crystallographic information

### Comment

Combining metal ions with oxadiazole-bridging organic ligands may result in coordination polymers with novel network connectivities (Dong *et al.*, 2003). Our interest in understanding the relationship between the metal coordination modes with such ligands and their extended structures led us to synthesize the title Hg^II^ compound, (I).

As shown in Fig. 1, there are five primary bonds to each Hg^II^ center, three Hg—N bonds and two Hg—Cl bonds, resulting in a distorted trigonal bipyramid coordination geometry around the Hg center. Three Hg—N bond distances (Table 1) are significantly different, but all agree with those reported previously (Gallagher *et al.*, 1999; Grupce *et al.*, 1999). The bond angles at Hg1 atom rang from 70.81 (18)° [N4—Hg—N3] to 145.19 (16)° [N4—Hg1—Cl1]. While the ligand chelates to a Hg atom by a pyridine N and an imine N atoms, the other pyridine N atom bridges to another Hg atom to form the title binuclear macrocyclic complex with the Hg···Hg separation of 12.969 (2) Å. Within the ligand, the dihedral angles between the oxadiazole and N4-pyridine rings and between the oxadiazole and N6-pyridine rings are identical [77.4 (4)°]. Intermolecular N—H···Cl and N—H···N hydrogen bonding helps to stabilize the crystal structure (Table 2).

### Experimental

2,5-Bis(4-aminophenyl)-1,3,4-oxadiazole (L1) was prepared according to the literature method (Ren *et al.*, 1995). A solution of L1 (2.56 g, 10 mmol) and 2-pyridylaldehyde (4 ml) in anhydrous EtOH (20 ml) was refluxed for 24 h, with HCOOH as catalyzer. After the mixture was cooled to room temperature, the precipitated product was filtered off, washed with EtOH and dried, yielding a light-yellow power [2,5-bis(4-((2-pyridinyl)methyleneamino)phenyl) -1,3,4-oxadiazole] (L2). Then the L2 was deoxidized by NaBH_4_ in anhydrous CH_3_OH (20 ml). The solvent was removed under reduced pressure, and the residue was washed with water to afford the ligand [2,5-bis(4-((2-pyridinylmethyl)amino)phenyl)-1,3,4-oxadiazole] (*L*) as a yellow solid. A solution of HgCl_2_ (13.58 mg, 0.05 mmol) in EtOH (8 ml) was layered onto a solution of the ligand *L* (21.7 mg, 0.05 mmol) in CH_2_Cl_2_ (8 ml). Single yellow crystals of the title compound were obtained after 7 d at room temperature.

### Refinement

All H atoms were placed in calculated positions with C—H = 0.93 (aromatic), 0.97 Å (methylene) and N—H = 0.91 or 0.86 Å imine groups), and refined using a riding model with *U*_iso_(H) = 1.2*U*_eq_(C,*N*).

### Crystal data

**Table:**

[Hg_2_Cl_4_(C_26_H_22_N_6_O)_2_]	*Z* = 1
*M**_r_* = 1411.98	*F*_000_ = 684
Triclinic, *P*1	*D*_x_ = 1.834 Mg m^−^^3^
Hall symbol: -P 1	Mo *K*α radiation λ = 0.71073 Å
*a* = 8.5426 (19) Å	Cell parameters from 2786 reflections
*b* = 9.945 (2) Å	θ = 2.5–25.6º
*c* = 16.533 (4) Å	µ = 6.26 mm^−^^1^
α = 83.773 (3)º	*T* = 298 (2) K
β = 80.001 (3)º	Block, yellow
γ = 67.671 (2)º	0.40 × 0.40 × 0.30 mm
*V* = 1278.2 (5) Å^3^	

### Data collection

**Table:**

Bruker SMART APEX CCD diffractometer	4652 independent reflections
Radiation source: fine-focus sealed tube	3878 reflections with *I* > 2σ(*I*)
Monochromator: graphite	*R*_int_ = 0.023
*T* = 298(2) K	θ_max_ = 25.5º
φ and ω scans	θ_min_ = 1.3º
Absorption correction: multi-scan(SADABS; Sheldrick, 2002)	*h* = −10→10
*T*_min_ = 0.113, *T*_max_ = 0.153	*k* = −12→11
6681 measured reflections	*l* = −20→12

### Refinement

**Table:**

Refinement on *F*^2^	Secondary atom site location: difference Fourier map
Least-squares matrix: full	Hydrogen site location: inferred from neighbouring sites
*R*[*F*^2^ > 2σ(*F*^2^)] = 0.044	H-atom parameters constrained
*wR*(*F*^2^) = 0.118	*w* = 1/[σ^2^(*F*_o_^2^) + (0.0677*P*)^2^ + 1.1058*P*] where *P* = (*F*_o_^2^ + 2*F*_c_^2^)/3
*S* = 1.04	(Δ/σ)_max_ = 0.001
4652 reflections	Δρ_max_ = 2.20 e Å^−^^3^
325 parameters	Δρ_min_ = −0.84 e Å^−^^3^
Primary atom site location: structure-invariant direct methods	Extinction correction: none

### Special details

**Table:**

Geometry. All e.s.d.'s (except the e.s.d. in the dihedral angle between two l.s. planes) are estimated using the full covariance matrix. The cell e.s.d.'s are taken into account individually in the estimation of e.s.d.'s in distances, angles and torsion angles; correlations between e.s.d.'s in cell parameters are only used when they are defined by crystal symmetry. An approximate (isotropic) treatment of cell e.s.d.'s is used for estimating e.s.d.'s involving l.s. planes.
Refinement. Refinement of *F*^2^ against ALL reflections. The weighted *R*-factor *wR* and goodness of fit *S* are based on *F*^2^, conventional *R*-factors *R* are based on *F*, with *F* set to zero for negative *F*^2^. The threshold expression of *F*^2^ > σ(*F*^2^) is used only for calculating *R*-factors(gt) *etc*. and is not relevant to the choice of reflections for refinement. *R*-factors based on *F*^2^ are statistically about twice as large as those based on *F*, and *R*- factors based on ALL data will be even larger.

### Fractional atomic coordinates and isotropic or equivalent isotropic displacement parameters (Å^2^)

**Table:**

	*x*	*y*	*z*	*U*_iso_*/*U*_eq_	
N6	0.7661 (9)	−0.1644 (7)	−0.4018 (4)	0.0529 (16)	
C5	0.6054 (9)	−0.1027 (8)	−0.3635 (4)	0.0418 (16)	
C6	0.5468 (11)	−0.2001 (8)	−0.2981 (4)	0.0516 (19)	
H6A	0.5341	−0.2764	−0.3251	0.062*	
H6B	0.6356	−0.2462	−0.2636	0.062*	
N5	0.3889 (8)	−0.1271 (7)	−0.2465 (4)	0.0495 (15)	
H5	0.2971	−0.1354	−0.2559	0.059*	
C7	0.3785 (9)	−0.0447 (7)	−0.1829 (4)	0.0399 (15)	
Hg1	0.95952 (4)	0.33183 (3)	0.324081 (19)	0.05155 (14)	
Cl1	1.0242 (3)	0.1191 (2)	0.25208 (17)	0.0723 (6)	
Cl2	0.6820 (3)	0.4107 (3)	0.41247 (15)	0.0854 (8)	
O1	0.4543 (5)	0.2726 (5)	0.0143 (3)	0.0354 (10)	
N3	0.8599 (7)	0.5232 (6)	0.2061 (3)	0.0371 (12)	
H3	0.9511	0.5157	0.1666	0.045*	
C19	0.7868 (8)	0.4113 (8)	0.1043 (4)	0.0413 (16)	
H19	0.9008	0.3795	0.0803	0.050*	
C13	0.3210 (8)	0.2390 (7)	0.0028 (4)	0.0380 (15)	
C14	0.3883 (8)	0.3660 (7)	0.0762 (4)	0.0356 (14)	
C18	0.7341 (8)	0.4991 (7)	0.1714 (4)	0.0363 (14)	
C9	0.4933 (9)	0.0866 (8)	−0.1119 (4)	0.0419 (16)	
H9	0.5837	0.1135	−0.1062	0.050*	
C11	0.2122 (8)	0.0950 (8)	−0.0669 (4)	0.0421 (16)	
H11	0.1104	0.1278	−0.0308	0.051*	
C10	0.3426 (8)	0.1393 (8)	−0.0594 (4)	0.0368 (14)	
N2	0.2294 (7)	0.3897 (7)	0.1005 (4)	0.0472 (15)	
C15	0.5034 (8)	0.4177 (7)	0.1072 (4)	0.0347 (14)	
C16	0.4485 (8)	0.5123 (8)	0.1708 (4)	0.0412 (16)	
H16	0.3331	0.5495	0.1924	0.049*	
C12	0.2300 (9)	0.0027 (8)	−0.1270 (4)	0.0436 (16)	
H12	0.1413	−0.0282	−0.1301	0.052*	
C21	0.8165 (10)	0.6524 (7)	0.2504 (5)	0.0481 (18)	
H21A	0.7151	0.6637	0.2903	0.058*	
H21B	0.7898	0.7365	0.2123	0.058*	
N1	0.1858 (7)	0.3049 (8)	0.0520 (4)	0.0508 (16)	
C23	1.0048 (10)	0.7665 (8)	0.2940 (5)	0.0516 (19)	
H23	0.9519	0.8514	0.2637	0.062*	
C20	0.6746 (8)	0.3714 (8)	0.0733 (4)	0.0404 (15)	
H20	0.7131	0.3121	0.0286	0.048*	
C4	0.5020 (11)	0.0367 (9)	−0.3836 (5)	0.0547 (19)	
H4	0.3909	0.0769	−0.3564	0.066*	
N4	1.0367 (7)	0.5233 (6)	0.3357 (3)	0.0420 (13)	
C22	0.9585 (9)	0.6475 (7)	0.2942 (4)	0.0401 (15)	
C25	1.2047 (10)	0.6336 (9)	0.3837 (5)	0.055 (2)	
H25	1.2881	0.6265	0.4151	0.066*	
C17	0.5620 (9)	0.5517 (8)	0.2022 (4)	0.0432 (16)	
H17	0.5226	0.6150	0.2451	0.052*	
C3	0.5663 (14)	0.1169 (10)	−0.4453 (6)	0.067 (2)	
H3A	0.5009	0.2133	−0.4581	0.080*	
C8	0.5139 (8)	−0.0050 (8)	−0.1727 (4)	0.0415 (16)	
H8	0.6178	−0.0405	−0.2071	0.050*	
C1	0.8213 (13)	−0.0863 (11)	−0.4622 (6)	0.069 (2)	
H1	0.9318	−0.1286	−0.4897	0.083*	
C24	1.1299 (11)	0.7583 (9)	0.3391 (5)	0.058 (2)	
H24	1.1628	0.8373	0.3391	0.069*	
C26	1.1544 (9)	0.5180 (8)	0.3812 (4)	0.0481 (17)	
H26	1.2039	0.4332	0.4124	0.058*	
C2	0.7255 (14)	0.0522 (11)	−0.4864 (6)	0.070 (3)	
H2	0.7688	0.1012	−0.5302	0.084*	

### Atomic displacement parameters (Å^2^)

**Table:**

	*U*^11^	*U*^22^	*U*^33^	*U*^12^	*U*^13^	*U*^23^
N6	0.064 (4)	0.042 (4)	0.052 (4)	−0.020 (3)	−0.011 (3)	0.011 (3)
C5	0.052 (4)	0.034 (4)	0.043 (4)	−0.017 (3)	−0.012 (3)	−0.006 (3)
C6	0.071 (5)	0.036 (4)	0.045 (4)	−0.019 (4)	−0.001 (4)	−0.003 (3)
N5	0.053 (4)	0.051 (4)	0.048 (4)	−0.023 (3)	−0.006 (3)	−0.012 (3)
C7	0.047 (4)	0.024 (3)	0.048 (4)	−0.013 (3)	−0.011 (3)	0.004 (3)
Hg1	0.0623 (2)	0.03300 (18)	0.0642 (2)	−0.02039 (14)	−0.02216 (14)	0.00926 (13)
Cl1	0.0659 (13)	0.0379 (11)	0.122 (2)	−0.0203 (10)	−0.0315 (12)	−0.0090 (11)
Cl2	0.0705 (14)	0.104 (2)	0.0654 (15)	−0.0260 (14)	−0.0016 (11)	0.0254 (13)
O1	0.030 (2)	0.039 (3)	0.039 (3)	−0.0149 (19)	−0.0056 (18)	−0.0019 (19)
N3	0.040 (3)	0.035 (3)	0.038 (3)	−0.016 (2)	−0.007 (2)	−0.001 (2)
C19	0.030 (3)	0.046 (4)	0.042 (4)	−0.009 (3)	0.000 (3)	−0.005 (3)
C13	0.036 (3)	0.039 (4)	0.041 (4)	−0.015 (3)	−0.011 (3)	0.004 (3)
C14	0.034 (3)	0.036 (4)	0.034 (4)	−0.010 (3)	−0.002 (3)	0.000 (3)
C18	0.041 (4)	0.034 (4)	0.036 (4)	−0.016 (3)	−0.008 (3)	0.004 (3)
C9	0.037 (4)	0.041 (4)	0.049 (4)	−0.016 (3)	−0.012 (3)	0.005 (3)
C11	0.034 (3)	0.048 (4)	0.043 (4)	−0.014 (3)	−0.005 (3)	−0.001 (3)
C10	0.035 (3)	0.042 (4)	0.036 (4)	−0.017 (3)	−0.012 (3)	0.004 (3)
N2	0.040 (3)	0.055 (4)	0.050 (4)	−0.020 (3)	0.002 (3)	−0.019 (3)
C15	0.031 (3)	0.037 (4)	0.035 (3)	−0.012 (3)	−0.007 (3)	0.006 (3)
C16	0.035 (3)	0.045 (4)	0.039 (4)	−0.011 (3)	−0.004 (3)	−0.004 (3)
C12	0.039 (4)	0.046 (4)	0.050 (4)	−0.021 (3)	−0.008 (3)	−0.002 (3)
C21	0.057 (4)	0.027 (4)	0.065 (5)	−0.013 (3)	−0.031 (4)	0.005 (3)
N1	0.035 (3)	0.070 (5)	0.055 (4)	−0.026 (3)	0.000 (3)	−0.022 (3)
C23	0.065 (5)	0.029 (4)	0.065 (5)	−0.014 (3)	−0.027 (4)	−0.001 (3)
C20	0.042 (4)	0.039 (4)	0.041 (4)	−0.015 (3)	−0.007 (3)	−0.005 (3)
C4	0.071 (5)	0.045 (5)	0.053 (5)	−0.022 (4)	−0.021 (4)	−0.001 (4)
N4	0.045 (3)	0.039 (3)	0.044 (3)	−0.013 (3)	−0.017 (3)	0.000 (3)
C22	0.049 (4)	0.029 (3)	0.040 (4)	−0.011 (3)	−0.011 (3)	−0.001 (3)
C25	0.057 (5)	0.055 (5)	0.059 (5)	−0.017 (4)	−0.027 (4)	−0.011 (4)
C17	0.045 (4)	0.041 (4)	0.042 (4)	−0.014 (3)	−0.003 (3)	−0.009 (3)
C3	0.094 (7)	0.042 (5)	0.071 (6)	−0.025 (5)	−0.040 (5)	0.017 (4)
C8	0.035 (3)	0.044 (4)	0.043 (4)	−0.010 (3)	−0.011 (3)	0.001 (3)
C1	0.072 (6)	0.076 (7)	0.062 (6)	−0.034 (5)	−0.014 (4)	0.014 (5)
C24	0.068 (5)	0.042 (5)	0.072 (5)	−0.023 (4)	−0.025 (4)	−0.009 (4)
C26	0.055 (4)	0.034 (4)	0.050 (4)	−0.006 (3)	−0.019 (3)	0.000 (3)
C2	0.096 (7)	0.077 (7)	0.060 (5)	−0.057 (6)	−0.032 (5)	0.028 (5)

### Geometric parameters (Å, °)

**Table:**

N6—C1	1.330 (11)	C11—C10	1.373 (9)
N6—C5	1.343 (10)	C11—C12	1.375 (10)
C5—C4	1.372 (11)	C11—H11	0.9300
C5—C6	1.520 (10)	N2—N1	1.407 (8)
C6—N5	1.437 (10)	C15—C16	1.385 (9)
C6—H6A	0.9700	C15—C20	1.388 (9)
C6—H6B	0.9700	C16—C17	1.368 (10)
N5—C7	1.373 (9)	C16—H16	0.9300
N5—H5	0.8600	C12—H12	0.9300
C7—C12	1.385 (10)	C21—C22	1.500 (10)
C7—C8	1.397 (9)	C21—H21A	0.9700
Hg1—Cl1	2.373 (2)	C21—H21B	0.9700
Hg1—Cl2	2.451 (2)	C23—C24	1.379 (11)
Hg1—N3	2.587 (6)	C23—C22	1.383 (10)
Hg1—N4	2.275 (6)	C23—H23	0.9300
Hg1—N6^i^	2.745 (7)	C20—H20	0.9300
O1—C13	1.350 (7)	C4—C3	1.394 (12)
O1—C14	1.355 (7)	C4—H4	0.9300
N3—C18	1.408 (8)	N4—C22	1.338 (9)
N3—C21	1.440 (9)	N4—C26	1.340 (9)
N3—H3	0.9100	C25—C24	1.361 (12)
C19—C20	1.358 (9)	C25—C26	1.378 (11)
C19—C18	1.390 (9)	C25—H25	0.9300
C19—H19	0.9300	C17—H17	0.9300
C13—N1	1.280 (9)	C3—C2	1.354 (14)
C13—C10	1.446 (9)	C3—H3A	0.9300
C14—N2	1.284 (8)	C8—H8	0.9300
C14—C15	1.452 (9)	C1—C2	1.365 (14)
C18—C17	1.381 (10)	C1—H1	0.9300
C9—C10	1.373 (10)	C24—H24	0.9300
C9—C8	1.374 (10)	C26—H26	0.9300
C9—H9	0.9300	C2—H2	0.9300
			
C1—N6—C5	117.3 (7)	C16—C15—C20	117.9 (6)
N6—C5—C4	122.1 (7)	C16—C15—C14	122.2 (6)
N6—C5—C6	114.8 (6)	C20—C15—C14	119.9 (6)
C4—C5—C6	123.0 (7)	C17—C16—C15	120.8 (6)
N5—C6—C5	114.9 (6)	C17—C16—H16	119.6
N5—C6—H6A	108.5	C15—C16—H16	119.6
C5—C6—H6A	108.5	C11—C12—C7	120.7 (6)
N5—C6—H6B	108.5	C11—C12—H12	119.6
C5—C6—H6B	108.5	C7—C12—H12	119.6
H6A—C6—H6B	107.5	N3—C21—C22	112.4 (6)
C7—N5—C6	122.8 (6)	N3—C21—H21A	109.1
C7—N5—H5	118.6	C22—C21—H21A	109.1
C6—N5—H5	118.6	N3—C21—H21B	109.1
N5—C7—C12	120.0 (6)	C22—C21—H21B	109.1
N5—C7—C8	121.8 (6)	H21A—C21—H21B	107.9
C12—C7—C8	118.1 (6)	C13—N1—N2	106.8 (5)
N4—Hg1—Cl1	145.19 (16)	C24—C23—C22	119.5 (7)
N4—Hg1—Cl2	99.31 (16)	C24—C23—H23	120.3
Cl1—Hg1—Cl2	114.87 (10)	C22—C23—H23	120.3
N4—Hg1—N3	70.81 (18)	C19—C20—C15	121.1 (6)
Cl1—Hg1—N3	98.44 (13)	C19—C20—H20	119.5
Cl2—Hg1—N3	95.30 (13)	C15—C20—H20	119.5
N4—Hg1—N6^i^	86.73 (19)	C5—C4—C3	118.9 (8)
Cl1—Hg1—N6^i^	84.60 (15)	C5—C4—H4	120.6
Cl2—Hg1—N6^i^	115.31 (15)	C3—C4—H4	120.6
N3—Hg1—N6^i^	144.82 (18)	C22—N4—C26	119.0 (6)
C13—O1—C14	103.7 (5)	C22—N4—Hg1	117.2 (4)
C18—N3—C21	120.2 (5)	C26—N4—Hg1	123.8 (5)
C18—N3—Hg1	110.2 (4)	N4—C22—C23	120.9 (7)
C21—N3—Hg1	98.4 (4)	N4—C22—C21	117.1 (6)
C18—N3—H3	109.1	C23—C22—C21	122.0 (6)
C21—N3—H3	109.1	C24—C25—C26	118.6 (7)
Hg1—N3—H3	109.1	C24—C25—H25	120.7
C20—C19—C18	121.1 (6)	C26—C25—H25	120.7
C20—C19—H19	119.5	C16—C17—C18	121.2 (6)
C18—C19—H19	119.5	C16—C17—H17	119.4
N1—C13—O1	111.7 (6)	C18—C17—H17	119.4
N1—C13—C10	128.3 (6)	C2—C3—C4	118.9 (8)
O1—C13—C10	120.0 (6)	C2—C3—H3A	120.6
N2—C14—O1	112.0 (6)	C4—C3—H3A	120.6
N2—C14—C15	130.1 (6)	C9—C8—C7	119.8 (6)
O1—C14—C15	117.8 (5)	C9—C8—H8	120.1
C17—C18—C19	117.8 (6)	C7—C8—H8	120.1
C17—C18—N3	124.0 (6)	N6—C1—C2	124.0 (9)
C19—C18—N3	118.1 (6)	N6—C1—H1	118.0
C10—C9—C8	121.6 (6)	C2—C1—H1	118.0
C10—C9—H9	119.2	C25—C24—C23	119.4 (7)
C8—C9—H9	119.2	C25—C24—H24	120.3
C10—C11—C12	120.9 (6)	C23—C24—H24	120.3
C10—C11—H11	119.5	N4—C26—C25	122.4 (7)
C12—C11—H11	119.5	N4—C26—H26	118.8
C11—C10—C9	118.5 (6)	C25—C26—H26	118.8
C11—C10—C13	120.4 (6)	C3—C2—C1	118.7 (9)
C9—C10—C13	121.0 (6)	C3—C2—H2	120.6
C14—N2—N1	105.8 (5)	C1—C2—H2	120.6
			
C1—N6—C5—C4	−1.7 (11)	C8—C7—C12—C11	4.7 (11)
C1—N6—C5—C6	177.0 (7)	C18—N3—C21—C22	170.7 (6)
N6—C5—C6—N5	168.7 (6)	Hg1—N3—C21—C22	51.3 (6)
C4—C5—C6—N5	−12.7 (11)	O1—C13—N1—N2	0.0 (8)
C5—C6—N5—C7	−77.9 (9)	C10—C13—N1—N2	−179.9 (7)
C6—N5—C7—C12	−169.2 (7)	C14—N2—N1—C13	−0.4 (8)
C6—N5—C7—C8	11.5 (11)	C18—C19—C20—C15	0.4 (11)
N4—Hg1—N3—C18	−162.2 (4)	C16—C15—C20—C19	3.5 (10)
Cl1—Hg1—N3—C18	52.0 (4)	C14—C15—C20—C19	−176.2 (6)
Cl2—Hg1—N3—C18	−64.2 (4)	N6—C5—C4—C3	−0.4 (11)
N6^i^—Hg1—N3—C18	144.6 (4)	C6—C5—C4—C3	−179.0 (7)
N4—Hg1—N3—C21	−35.6 (4)	Cl1—Hg1—N4—C22	93.9 (5)
Cl1—Hg1—N3—C21	178.6 (4)	Cl2—Hg1—N4—C22	−75.3 (5)
Cl2—Hg1—N3—C21	62.4 (4)	N3—Hg1—N4—C22	17.1 (5)
N6^i^—Hg1—N3—C21	−88.8 (5)	N6^i^—Hg1—N4—C22	169.6 (5)
C14—O1—C13—N1	0.3 (8)	Cl1—Hg1—N4—C26	−86.1 (6)
C14—O1—C13—C10	−179.8 (6)	Cl2—Hg1—N4—C26	104.7 (5)
C13—O1—C14—N2	−0.6 (7)	N3—Hg1—N4—C26	−162.9 (6)
C13—O1—C14—C15	176.6 (6)	N6^i^—Hg1—N4—C26	−10.5 (6)
C20—C19—C18—C17	−3.9 (11)	C26—N4—C22—C23	3.9 (10)
C20—C19—C18—N3	173.1 (6)	Hg1—N4—C22—C23	−176.2 (5)
C21—N3—C18—C17	−28.4 (10)	C26—N4—C22—C21	−174.5 (6)
Hg1—N3—C18—C17	84.8 (7)	Hg1—N4—C22—C21	5.4 (8)
C21—N3—C18—C19	154.7 (7)	C24—C23—C22—N4	−1.9 (11)
Hg1—N3—C18—C19	−92.1 (6)	C24—C23—C22—C21	176.4 (7)
C12—C11—C10—C9	−1.6 (11)	N3—C21—C22—N4	−44.7 (9)
C12—C11—C10—C13	178.6 (7)	N3—C21—C22—C23	136.9 (7)
C8—C9—C10—C11	2.0 (11)	C15—C16—C17—C18	0.3 (11)
C8—C9—C10—C13	−178.2 (6)	C19—C18—C17—C16	3.6 (11)
N1—C13—C10—C11	−5.6 (12)	N3—C18—C17—C16	−173.3 (6)
O1—C13—C10—C11	174.5 (6)	C5—C4—C3—C2	3.3 (12)
N1—C13—C10—C9	174.6 (8)	C10—C9—C8—C7	0.9 (11)
O1—C13—C10—C9	−5.3 (10)	N5—C7—C8—C9	175.1 (7)
O1—C14—N2—N1	0.6 (8)	C12—C7—C8—C9	−4.3 (10)
C15—C14—N2—N1	−176.1 (7)	C5—N6—C1—C2	0.9 (13)
N2—C14—C15—C16	−2.9 (12)	C26—C25—C24—C23	0.9 (13)
O1—C14—C15—C16	−179.4 (6)	C22—C23—C24—C25	−0.6 (12)
N2—C14—C15—C20	176.7 (7)	C22—N4—C26—C25	−3.5 (11)
O1—C14—C15—C20	0.2 (9)	Hg1—N4—C26—C25	176.6 (6)
C20—C15—C16—C17	−3.8 (10)	C24—C25—C26—N4	1.1 (12)
C14—C15—C16—C17	175.8 (7)	C4—C3—C2—C1	−4.1 (13)
C10—C11—C12—C7	−1.8 (11)	N6—C1—C2—C3	2.0 (15)
N5—C7—C12—C11	−174.7 (7)		

Symmetry codes: (i) −*x*+2, −*y*, −*z*.

### Hydrogen-bond geometry (Å, °)

**Table:**

*D*—H···*A*	*D*—H	H···*A*	*D*···*A*	*D*—H···*A*
N3—H3···N2^ii^	0.91	2.36	3.191 (8)	152
N5—H5···Cl1^iii^	0.86	2.68	3.517 (7)	166

Symmetry codes: (ii) *x*+1, *y*, *z*; (iii) −*x*+1, −*y*, −*z*.
